# ENGAGING WITH MUNICIPAL PLANNERS WITH A DEMENTIA-INCLUSIVE PLANNING AND DESIGN GUIDE

**DOI:** 10.1093/geroni/igae098.1334

**Published:** 2024-12-31

**Authors:** Kishore Seetharaman, Habib Chaudhury

**Affiliations:** Simon Fraser University, Vancouver, British Columbia, Canada; Simon Fraser University, Vancouver, British Columbia, Canada

## Abstract

Dementia-friendly and inclusive community (DFC) action plans in British Columbian municipalities identify various planning and design strategies and actions to meet the goals of dementia-inclusivity. However, there is a lack of evidence-based design guidelines tailored to the context of British Columbia to support the implementation of these plans. To address this gap, a DFC planning and design guide was generated as part of the DemSCAPE study, consisting of planning and design principles and guidelines for wayfinding, safety, social interaction and access to amenities for people living with dementia. A series of knowledge mobilization (KM) activities will explore and augment the relevance, uptake, and implementation of this guide. Two focus groups (60-90 min; N = 5-7 per session) were conducted with municipal professionals working in two municipalities (i.e., City of Burnaby and City of Richmond) in Metro Vancouver. The existing municipal action plans emphasize the need for collaboration and dialogue across different departments of local government, e.g., social planners and seniors services coordinators (staff who are more closely connected with and help coordinate these plans) and planners, designers, and engineers. The focus groups are intended to trigger inter-departmental dialogue centered on DFC planning and design and examine DFC strategies and actions from various stakeholders’ perspectives within the scope of municipal planning. This KM project is expected to make meaningful contributions to the DFC initiatives of local government in BC by providing guidelines to municipal planners on how to apply a dementia-friendly and inclusive lens in planning and design.

